# Supplementary material for: Preparation, Characterization, and Evaluation of Liposomes Containing Oridonin from Rabdosia rubescens
Source: Molecules. 2022 Jan 27;27(3):860. doi: 10.3390/molecules27030860 (PMC8839758; doi:10.3390/molecules27030860)
Supplement: Supplementary file 1 [file molecules-27-00860-s001.zip › molecules-1535779-supplementary.pdf]

Table S1. Analysis of variance (ANOVA) for the response quadratic model.

Effects	Source	Sum of squares	Degree of freedom (DF)	Mean square	F value	*p*- value
	model	1.12	9	0.1246	26.31	0.0001[a]
Linear	X_1	0.5279	1	0.5279	111.43	< 0.0001 [a]
	X_2	0.0229	1	0.0229	4.83	0.0640 [b]
	X_3	0.0000	1	0.0000	0.0031	0.9573 [b]
Interaction	X_1X_2	0.0869	1	0.0869	18.34	0.0036 [a]
	X_1X_3	0.1095	1	0.1095	23.12	0.0019 [a]
	X_2X_3	0.0047	1	0.0047	0.9947	0.3518 [b]
Quadratic	X_1^2	0.1934	1	0.1934	40.82	0.0004 [a]
	X_2^2	0.0909	1	0.0909	19.19	0.0032 [a]
	X_3^2	0.0502	1	0.0502	10.59	0.0140 [a]
	Residual	0.83	5	0.17		
	Lack of fit	0.78	3	0.26	9.49	0.0968[b]
	Pure error	0.054	2	0.027		
	Cor. total	1.15	14			

R^2=0.9713, adjusted R^2=0.9344 C.V.%=1.77%. [a] 5% significance level. [b]Not significant relative to the pure error.
